# Antibody-dependent enhancement of porcine reproductive and respiratory syndrome virus infection downregulates the levels of interferon-gamma/lambdas in porcine alveolar macrophages *in vitro*

**DOI:** 10.3389/fvets.2023.1150430

**Published:** 2023-03-15

**Authors:** Liujun Zhang, Xing Feng, Huandi Wang, Shaojun He, Hongjie Fan, Deyi Liu

**Affiliations:** College of Animal Science, Anhui Science and Technology University, Chuzhou, China

**Keywords:** PRRSV-ADE, IFN-γ, IFN-λs, TGF-β1, PAMs

## Abstract

Fc gamma receptor-mediated antibody-dependent enhancement (ADE) can promote virus invasion of target cells, sometimes exacerbating the severity of the disease. ADE may be an enormous hurdle to developing efficacious vaccines for certain human and animal viruses. ADE of porcine reproductive and respiratory syndrome virus (PRRSV) infection has been demonstrated *in vivo* and *in vitro*. However, the effect of PRRSV-ADE infection on the natural antiviral immunity of the host cells is yet to be well investigated. Specifically, whether the ADE of PRRSV infection affects the levels of type II (interferon-gamma, IFN-γ) and III (interferon-lambdas, IFN-λs) interferons (IFNs) remains unclear. In this study, our results showed that PRRSV significantly induced the secretion of IFN-γ, IFN-λ1, IFN-λ3, and IFN-λ4 in porcine alveolar macrophages (PAMs) in early infection, and weakly inhibited the production of IFN-γ, IFN-λ1, IFN-λ3, and IFN-λ4 in PAMs in late infection. Simultaneously, PRRSV infection significantly increased the transcription of interferon-stimulated gene 15 (ISG15), ISG56, and 2′, 5′-oligoadenylate synthetase 2 (OAS2) in PAMs. In addition, our results showed that PRRSV infection in PAMs *via* the ADE pathway not only significantly decreased the synthesis of IFN-γ, IFN-λ1, IFN-λ3, and IFN-λ4 but also significantly enhanced the generation of transforming growth factor-beta1 (TGF-β1). Our results also showed that the ADE of PRRSV infection significantly reduced the mRNAs of ISG15, ISG56, and OAS2 in PAMs. In conclusion, our studies indicated that PRRSV-ADE infection suppressed innate antiviral response by downregulating the levels of type II and III IFNs, hence facilitating viral replication in PAMs *in vitro*. The ADE mechanism demonstrated in the present study furthered our understanding of persistent pathogenesis following PRRSV infection mediated by antibodies.

## Introduction

Porcine reproductive and respiratory syndrome (PRRS) is a highly contagious disease of pigs, which first emerged in North America in the late 1980s and subsequently in Western Europe in the early 1990s (1). PRRS is a significant threat to swine health. It is best characterized by severe respiratory disorders and high mortality in piglets, preterm birth, fetal death, late-term abortions, and mummified fetuses in pregnant sows, resulting in devastating economic losses to the modern pig industry worldwide for over three decades (2, 3). PRRS virus (PRRSV), as the etiological agent of this disease, is an enveloped virus containing one single-stranded positive RNA genome of ~15.4 kb in length. This virus, together with the equine arteritis virus, simian hemorrhagic fever virus, and mouse lactate dehydrogenase-elevating virus, belongs to the Arteriviridae family within the order Nidovirales (4). All PRRSV strains are grouped into two species: PRRSV-1 (European or type 1 genotype) and PRRSV-2 (North American or type 2 genotype). Interestingly, although both genotypes show only ~60% identity at the nucleotide levels of genomic sequences with a high degree of antigenic divergence, they lead to similar clinical manifestations in infected pigs (5, 6). Swine are the only known natural hosts of PRRSV. The porcine monocyte-macrophage lineage, particularly monocyte-derived inflammatory dendritic cells, and differentiated macrophages, such as porcine alveolar macrophages (PAMs), are the PRRSV principal permissive cells (7, 8). In addition, the immortalized monkey kidney epithelial cell line, Marc-145 cells, can be infected by PRRSV (9).

Antibody-dependent enhancement (ADE), an immunopathological phenomenon in which preexisting specific non- or sub-neutralizing antibodies enhance the entry and replication of the virus in the host cells, has been described for many viruses, including PRRSV (10). As early as 1993, Christianson et al. reported that PRRSV infectivity increased in fetuses by adding porcine serum containing PRRSV antibodies (11). Later, Yoon et al. directly revealed the presence of PRRSV-ADE *in vivo* by injecting swine with PRRSV-specific antibodies. The duration of viremia is more significant in pigs injected with sub-neutralizing anti-PRRSV immunoglobulin G (IgG) before the virus challenge than in control pigs injected with normal IgG (12). The prolonged duration of viremia and the isolation of the virus from the tissues of piglets with low maternal antibodies ulteriorly provide evidence of *in vivo* PRRSV-ADE activity (13). *In vitro*, the enhanced replication of PRRSV in the presence of sub-neutralizing specific antibodies against PRRSV has also been confirmed (14, 15). These studies show that ADE is likely to increase the severity of PRRS and the susceptibility to PRRSV in pigs with declining maternal antibodies for PRRSV or with low levels of specific antibodies induced by exposure to wild-type or vaccine virus strains. Currently, commercially available PRRS vaccines provide insufficient protection against PRRSV, especially emerging and heterologous field virus strains (16–18). The immunological effect of PRRS vaccines is impacted by multitudinous host factors, in which ADE may be one crucial factor (19, 20). Vaccine-induced enhancement of susceptibility to virus infection or aberrant viral pathogenesis is also a significant obstacle in developing certain paramyxo-, corona-, flavi-, and lentivirus vaccines (21, 22). Therefore, more effective prevention and control strategies for PRRSV are still needed. A clear understanding of the ADE event could prevent the disease.

The innate immune response is the first line of host protection against viral infections. The suppression of intracellular antiviral immune signals by the virus using the ADE mechanism plays a vital role in the pathogenesis of persistent infection of the disease (23–25). PRRSV modulates host innate antiviral immunity by modifying the expression patterns of crucial antiviral cytokines such as interferons (IFNs) (26, 27). However, the roles of PRRSV-ADE in the natural immune defense system of the host are not well understood. In this study, we assessed the effect of PRRSV-ADE infection on the production of type II (IFN-γ) and III (IFN-λ1, IFN-λ3, and IFN-λ4) IFNs, and surveyed the transcription expression of several critical downstream antiviral protein genes (interferon-stimulated gene 15, ISG15; ISG56; and 2′, 5′-oligoadenylate synthetase 2, OAS2). The current study suggests that PRRSV infection suppressed IFN-γ/λs antiviral responses *via* the antibody-dependent pathway in PAMs *in vitro*. These results would help facilitate the understanding of PRRSV-persistent pathogenesis and antiviral vaccination strategies.

## Materials and methods

### Cells

Marc-145 cells maintained in Dulbecco's modified Eagle's medium (Sangon Biotech, Shanghai, China) supplemented with 10% fetal calf serum (FCS) (Tianhang Biotech, Huzhou, China) were used to propagate PRRSV and titrate its 50% tissue culture infectious dose (TCID_50_) employing the Reed–Muench method. PAMs derived from 3 to 6-week-old PRRSV-negative piglets using lung lavage were cultivated in a Roswell Park Memorial Institute (RPMI)-1640 medium (Sangon Biotech) containing 10% FCS plus 100 μg/ml streptomycin and 100 U/ml penicillin (Sangon Biotech). These two cells were kept in a humidified atmosphere with 5% CO_2_ at 37°C before use.

### Virus and antibodies

The type 2 PRRSV HeN-3 strain used in the current study was a kind gift from Prof. Pingan Xia of Henan Agricultural University. The inactivated purified HeN-3 virus particles were used to immunize pigs to produce PRRSV-positive sera (Enzyme-linked immunosorbent assay (ELISA) titer: 6400). PRRSV-negative serum samples were collected from PRRSV-negative healthy piglets. The IgG antibodies were depurated using diethyl-aminoethanol chromatography.

### Preparation of infectious PRRSV–antibody complexes

Approximately 2000 TCID_50_/ml of PRRSV was fully incubated with an equal volume of 850 μg/ml of purified PRRSV-positive IgG (PPI) or PRRSV-negative IgG (PNI) for 1 h at 37°C to yield infectious virus–antibody complexes (PRRSV+PPI) or controls (PRRSV+PNI), respectively.

### PRRSV or PRRSV-ADE infection in PAMs

Six hours before infection, PAMs were dispensed into 24-well plates (Sangon Biotech) at a density of 5 × 10^5^ cells/well. After discarding the culture solutions and washing the cells gently three times with FCS-free RPMI-1640, 200 μl poly (I:C) (100 μg/ml) (Sigma, Missouri, USA), PRRSV (200 TCID_50_), PRRSV+PNI, or PRRSV+PPI was inoculated onto the cell monolayers at 37°C for 2 h. Then, the inoculum was removed, and 500 μl fresh growth media was added. The uninfected cells served as mock trials. The cells of each well and culture supernatants were harvested every 12 h post-infection for real-time RT-PCR (28), virus titration, relative quantitative RT-PCR, or ELISA assay.

### Relative quantitative RT-PCR

Total RNA from PAMs was extracted using TRIzol reagent (Takara Bio, Beijing, China), and cDNAs were synthesized using RT reagent Kits (Takara Bio). Subsequently, the cDNAs were used for the amplification of relative quantitative RT-PCR. The primer pairs are described in Table 1. The PCR reaction volume was 20 μl and comprised 10 μl TB Green^®^ Premix Ex Taq™ II (Tli RNaseH Plus) (Takara Bio), 2 μl forward and reverse primers (20 pmol/μl), 2 μl cDNA template, and 6 μl sterile double-distilled water. The relative quantitative RT-PCR was performed on a QuantStudio5 Real-Time PCR System (Applied Biosystems, Massachusetts, USA). The thermocycling conditions were 95°C for 5 min, 95°C for 5 s, and 60°C for 34 s with 40 cycles. The 2${}_{\text{T}}^{-\text{Δ}\Delta C}$ method was adopted to analyze the quantification of the target genes.

**Table 1:** Primers used for the relative quantitative RT-PCR.

**Name^#^**	**Sequence (5^′^-3′)**
IFN-γ F	AGCCAAATTGTCTCCTTCTA
IFN-γ R	AAGTCATTCAGTTTCCCAGA
IFN-λ1 F	AACTTCAGGCTTGCATCAGG
IFN-λ1 R	TCTTTCTTTGTGGCTTCTTGG
IFN-λ3 F	TTGGAGGACTGGAACTGC
IFN-λ3 R	AGCTGGGCGTGGATGTG
IFN-λ4 F	GTGGCTATGGGACTGTGGG
IFN-λ4 R	TCCAGGGAGCGGTAGTGAG
TGF-β1 F	GAGCCAGAGGCGGACTA
TGF-β1 R	GGGTGCCCTTGAATTTATC
ISG15 F	GGTGCAAAGCTTCAGAGACC
ISG15 R	GTCAGCCAGACCTCATAGGC
ISG56 F	TCAGAGGTGAGAAGGCTGGT
ISG56 R	GCTTCCTGCAAGTGTCCTTC
OAS2 F	CACAGCTCAGGGATTTCAGA
OAS2 R	TCCAACGACAGGGTTTGTAA
β-actin F	CGGGACATCAAGGAGAAGC
β-actin R	CTCGTTGCCGATGGTGATG

^#^F, forward primer; R, reverse primer; The ISG15, ISG56, and OAS2 primers were quoted from reference (29).

### ELISA assay

The protein concentrations of IFN-γ, IFN-λ1, IFN-λ3, IFN-λ4, and TGF-β1 in the cell culture supernatants were detected using commercial ELISA Kits according to the manufacturer's protocols. The ELISA Kits for IFN-γ or TGF-β1 were purchased from R&D Systems in the United States (Minnesota). MyBioSource Inc. (California, USA) provided the IFN-λ1, IFN-λ3, and IFN-λ4 ELISA Kits.

### Statistical analysis

The statistical analyses were conducted using a two-way analysis of variance (ANOVA) followed by Bonferroni post-tests using GraphPad Prism 5.0 (GraphPad Software Inc.). A *p*-value of < 0.05 was considered significant.

## Results

### Presence of PRRSV-ADE activity in swine anti-PRRSV antibodies (IgGs)

**Figure 1:**
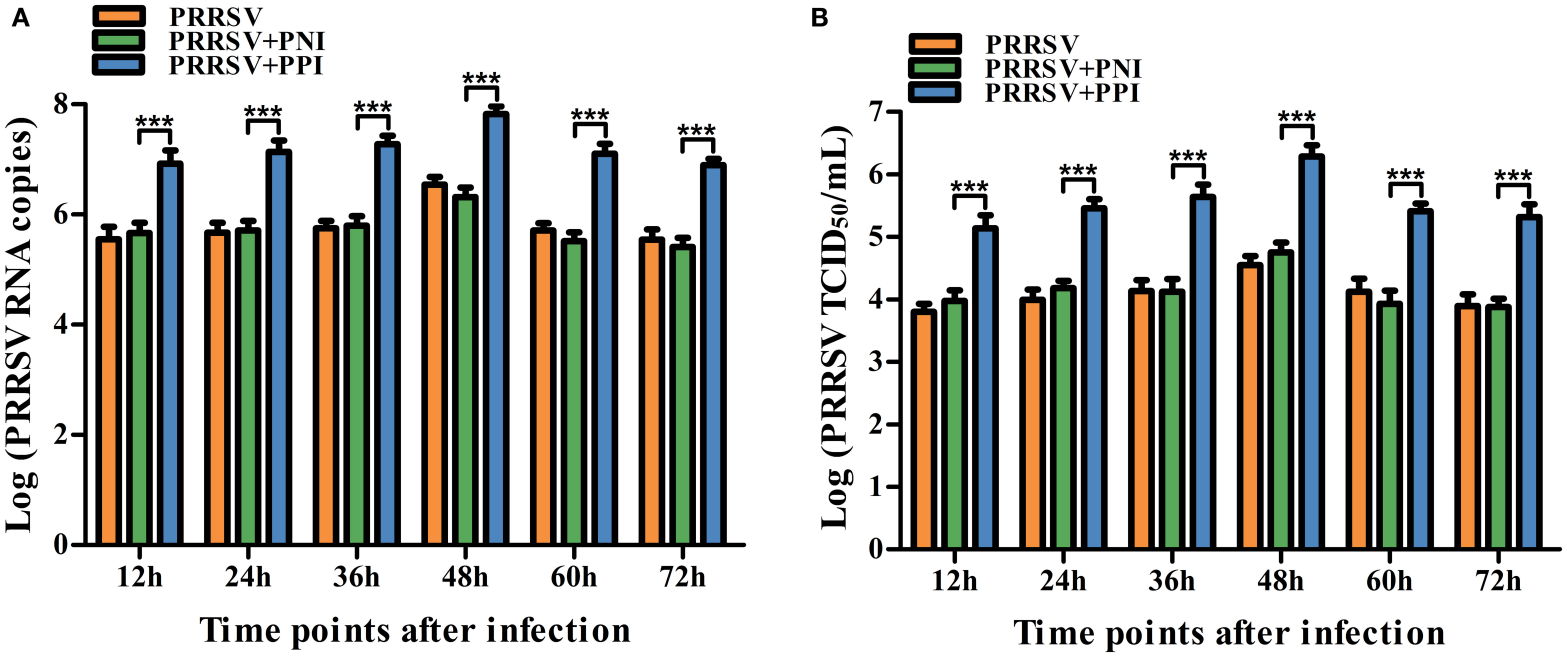
Detection of PRRSV-ADE activity in PAMs. PRRSV RNA **(A)** and TCID_50_ **(B)** in harvested culture supernatants of the PAM cells following PRRSV, PRRSV+PPI, or PRRSV+PNI infection were measured using real-time RT-PCR and viral titration experiments. The bars indicate the RNA copies or TCID_50_ titers of PRRSV. The error bars indicate the mean ± standard error of the mean (SEM) from three independent experiments. ***p < 0.001.

To confirm whether swine anti-PRRSV specific antibodies mediated the ADE phenomenon, PRRSV-positive IgG (PPI) and PRRSV-negative IgG (PNI) originating from pigs were tested for the activity of ADE infection by comparing their ability to enhance PRRSV replication in PAMs. Approximately 850 μg/ml of the purified PPI or PNI was mixed with 2,000 TCID_50_/ml of PRRSV in equal volumes and incubated at 37°C for 1 h to accelerate the generation of infectious virus–antibody immune complexes (PRRSV+PPI) or control groups (PRRSV+PNI). The PAM cell monolayers seeded into 24-well plates were infected with PRRSV, PRRSV+PNI, or PRRSV+PPI for the indicated time points. The infected cell supernatants were collected for demonstration of the yields of the virus by measuring the RNA and TCID_50_ of PRRSV. The results are shown in Figure 1. Compared with the control group cells infected with PRRSV+PNI, clear and significant increases (*p* < 0.001) in both PRRSV RNA and TCID_50_ were observed in cell culture supernatants of PAMs infected with PRRSV+PPI for 12–72 h. The increases in viral RNA and TCID_50_ in PAMs mediated by PPI ranged from 18.18 to 38.88 folds and 14.39–34.22 folds, respectively. By contrast, the kinetics of viral infectivity in PRRSV +PNI and PRRSV alone infected cells were similar. These results suggest that PPI facilitates the proliferation of PRRSV in PAMs. Therefore, ADE activity exists in swine anti-PRRSV antibodies.

### PRRSV induces IFN-γ/λs antiviral responses in PAMs

**Figure 2:**
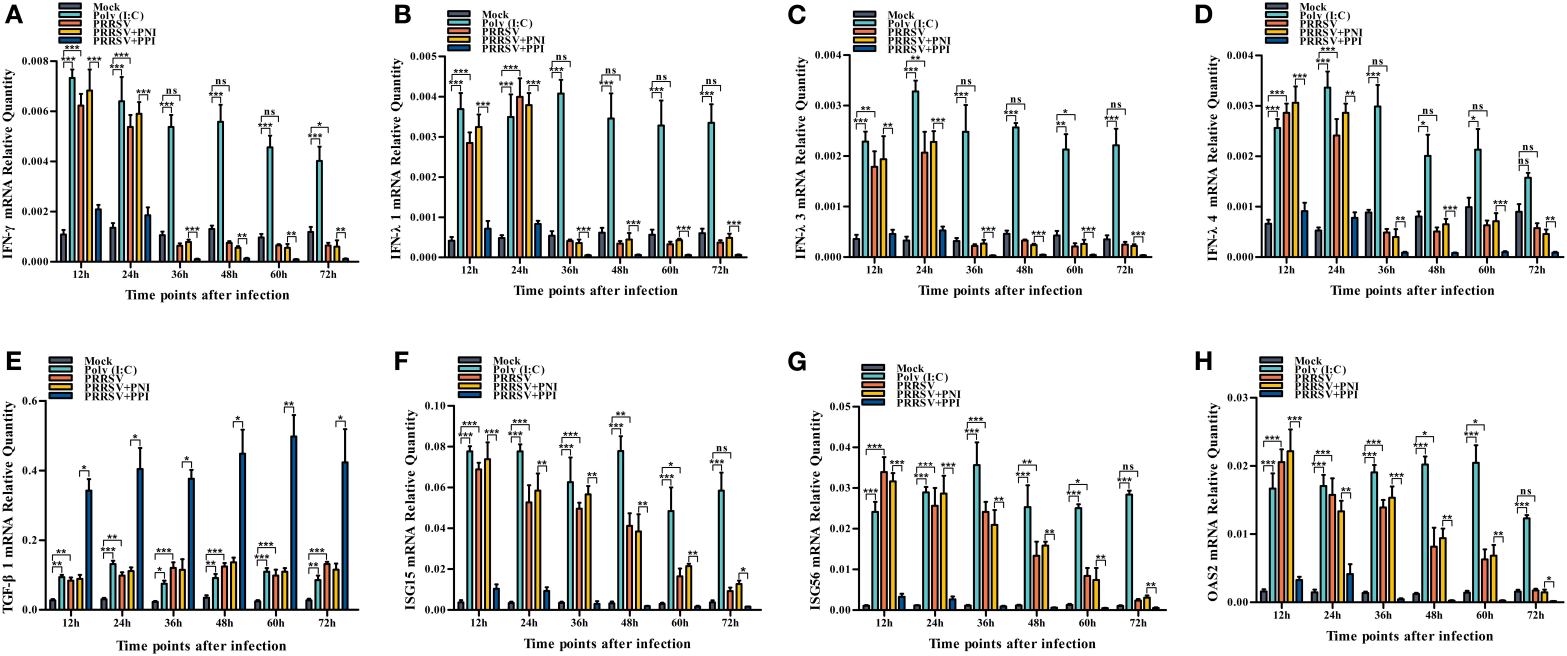
The effect of PRRSV or PRRSV-ADE on mRNAs of the innate immune cytokines and antiviral protein genes in PAMs. The mRNAs of the innate immune cytokines and antiviral protein genes in treated PAM cells were evaluated using relative quantitative RT-PCR. **(A)** IFN-γ mRNA, **(B)** IFN-λ1 mRNA, **(C)** IFN-λ3 mRNA, **(D)** IFN-λ4 mRNA, **(E)** TGF-β1 mRNA, **(F)** ISG15 mRNA, **(G)** ISG56 mRNA, and **(H)** OSA2 mRNA. The bars indicate the relative expression levels of mRNAs of the innate immune cytokines or antiviral protein genes. The error bars indicate the SEM from three independent experiments. ***p < 0.001, **p < 0.01, *p < 0.05, and ns, no significance.

**Figure 3:**
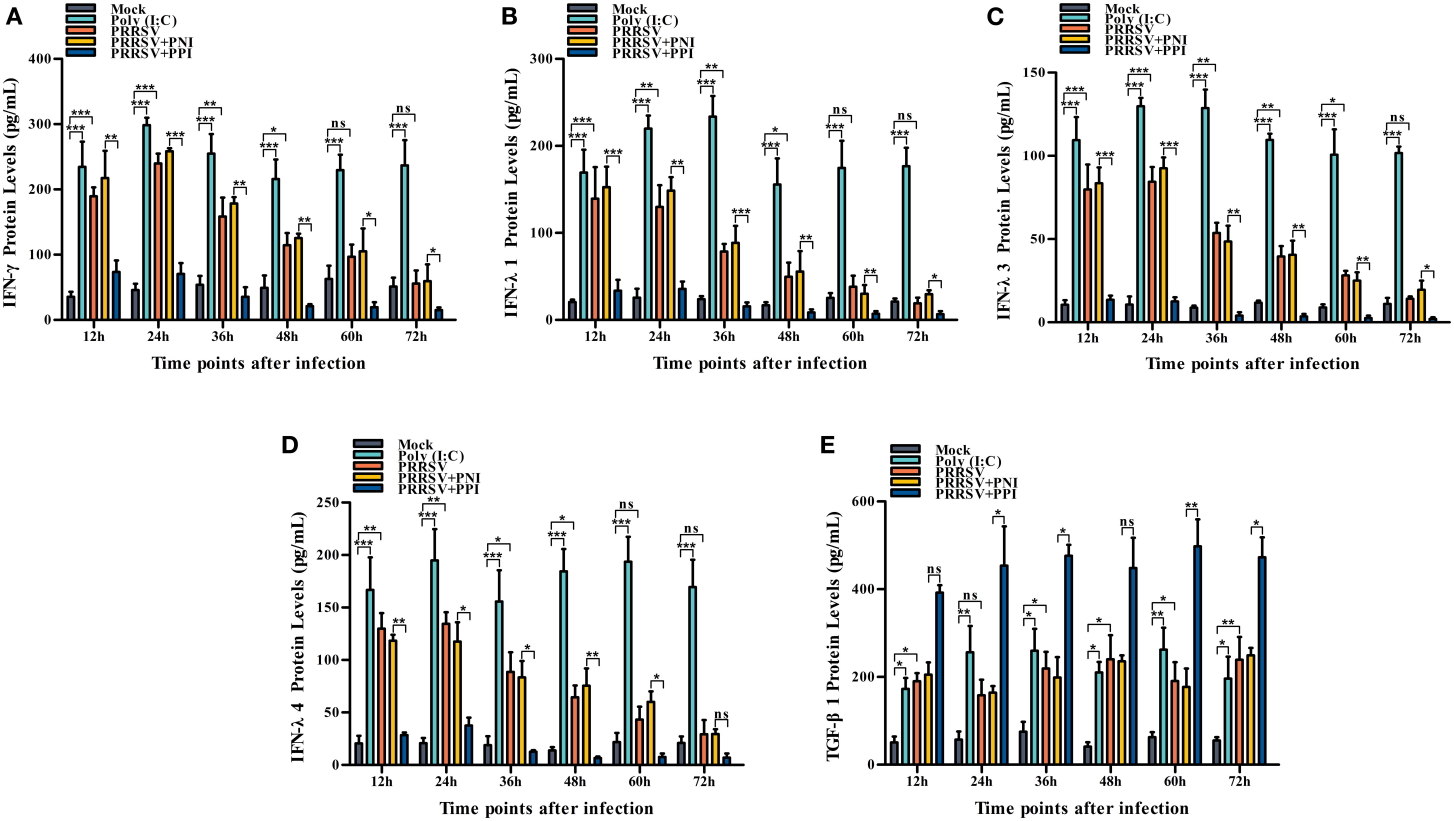
The effect of PRRSV or PRRSV-ADE on proteins of the innate immune cytokines in PAMs. The proteins of the innate immune cytokines in the culture supernatants of the PAM cells treated with the indicated methods were quantified using commercial ELISA Kits. **(A)** IFN-γ protein, **(B)** IFN-λ1 protein, **(C)** IFN-λ3 protein, **(D)** IFN-λ4 protein, and **(E)** TGF-β1 protein. The bars indicate the protein concentrations of the innate immune cytokines. The error bars indicate the SEM from three independent experiments. ***p < 0.001, **p < 0.01, *p < 0.05, and ns, no significance.

Several key innate immune cytokines (IFN-γ, IFN-λ1, IFN-λ3, IFN-λ4, and TGF-β1) and downstream antiviral protein genes (ISG15, ISG56, and OAS2) closely associated with viral replication were selected for post-PRRSV infection investigation to determine the effect of the virus on innate antiviral response in PAMs. In the case of PRRSV infection alone, the transcripts of IFN-γ, IFN-λ1, IFN-λ3, and IFN-λ4 in the infected PAM cells increased remarkably at 12–24 h postinfection and decreased slightly at 36–72 h postinfection (Figures 2A–D). Additionally, the protein concentrations of IFN-γ, IFN-λ1, IFN-λ3, and IFN-λ4 in the infected cell culture supernatants were significantly enhanced by PRRSV at 12–48 h postinfection, displaying rapid decline with the duration of virus infection (Figures 3A–D). Nevertheless, the data in Figures 2E, 3E show that the mRNA and protein concentration of TGF-β1 in the PAM cells increased observably in the case of infection with PRRSV for 12–72 h. Furthermore, relative quantitative analysis shows that the transcriptional levels of ISG15, ISG56, and OAS2 in cells infected with PRRSV for 12–60 h were signally heightened compared with the untreated mock control group cells (Figures 2F–H). These results suggest that PRRSV infection induces IFN-γ/λs antiviral responses in PAMs.

### PRRSV-ADE represses IFN-γ/λs antiviral responses in PAMs

We demonstrated that PRRSV could activate the IFN-γ/λs antiviral responses of host cells. We further examined whether the ADE of PRRSV infection also affected the innate antiviral response by comparing the mRNA or protein levels of IFN-γ, IFN-λ1, IFN-λ3, IFN-λ4, TGF-β1, ISG15, ISG56, and OAS2 in PAMs cells treated with PRRSV+PNI or PRRSV+PPI for the indicated time points. The results obtained from the relative quantitative RT-PCR and ELISA assay show that the expression profiles of IFN-γ, IFN-λ1, IFN-λ3, IFN-λ4, TGF-β1, ISG15, ISG56, and OAS2 in PRRSV+PNI- and PRRSV+PPI-treated macrophages are similar (Figures 2, 3). However, there is a significant downregulation of the transcriptional levels and protein production of IFN-γ, IFN-λ1, IFN-λ3, and IFN-λ4 in PRRSV+PPI-infected macrophages at 12–72 h postinfection compared with PRRSV+PNI cultures (Figures 2A–D, 3A–D). By contrast, the TGF-β1 mRNA and its protein levels were significantly upregulated in the macrophages following the PRRSV+PPI infection (Figures 2E, 3E). Moreover, as summarized in Figures 2F–H, the quantitative analysis shows that the amounts of ISG15, ISG56, and OAS2 mRNA are reduced significantly in the PAM cells infected with PRRSV+PPI for any time point compared with the PRRSV+PNI-infected PAM cells. These results suggest that PRRSV-ADE infection represses IFN-γ/λs antiviral responses in PAMs.

## Discussion

IFNs have become essential to the natural immune system against viruses by evoking potent early antiviral responses and regulating subsequent adaptive immunity (30). Three types of IFNs have been described in the IFN family, namely types I–III. Type II IFN only contains a single IFN-γ. Conversely, both type I and III IFNs are composed of multiple subtypes. For example, the groups of type I IFNs include IFN-α, IFN-β, IFN-ω, IFN-ε, and IFN-κ. The recently discovered type III IFNs, also called IFN-λs, consist of three members in pigs (IFN-λ1, IFN-λ3, and IFN-λ4), two in mice (IFN-λ2 and IFN-λ3), and four in humans (IFN-λ1, IFN-λ2, IFN-λ3, and IFN-λ4) (31, 32). The binding of IFNs to their corresponding receptors, with ensuing downstream signal transduction, triggers the expression of a series of ISGs, such as ISG15, ISG56, and OAS2. These ISGs mediate the antiviral effects of IFNs (33). There is conflicting evidence regarding the ability of PRRSV to induce IFN responses both *in vivo* and *in vitro* (27, 34, 35). Further research has shown that different PRRSV strains differ in their capability to induce IFNs (36, 37). We found that after infection with the PRRSV HeN-3 strain, PAMs secreted appreciable levels of IFN-γ, IFN-λ1, IFN-λ3, and IFN-λ4 in the early stage of infection. Furthermore, a significant increase was observed in the mRNA of antiviral genes ISG15, ISG56, and OAS2 in the PRRSV-infected PAMs compared with the mock-treated PAMs. However, due to the lack of available antibodies, we could not quantify the protein levels of these antiviral genes. Our current results collectively demonstrate that PRRSV can induce IFN-γ/λs antiviral responses in its host cells. IFN regulatory factor-3 (IRF-3), IRF-7, and nuclear factor kappa-B (NF-κB) are critical transcription factors of IFN production (32, 38). The results in Supplementary Figures 1A, B show that the mRNA levels of IRF-3, IRF-7, and NF-κB in PAMs are considerably upregulated by PRRSV at 12–24 h postinfection. PRRSV may activate IFN-γ/λs production by upregulating the levels of these transcription factors. IFNs are not the only cytokines affected by PRRSV. TGF-β1 is a negative immune regulator that may influence virus replication (39). We found that PRRSV enhanced the transcription and protein levels of TGF-β1 in PAMs, consistent with previous reports (40, 41).

Halstead and O'Rourke first suggested the FcγR-mediated ADE mechanism of virus infection in 1977 (42). Macrophages are immune cells and important host cells of various respiratory pathogens. ADE may promote the viral entry into macrophages through Fc receptor-mediated endocytosis and alter the endocellular signaling pathways, leading to their switching from an antiviral mode to a viral susceptibility mode (25, 43, 44). Data from several early studies show that there was a downregulation of type I (IFN-α/β) and II (IFN-γ) IFNs during ADE infection of the dengue virus or Ross river virus (45–47). A strong suppression of type I IFNs, including IFN-α and –β, was also reported in the ADE of PRRSV infection (29, 48). Whether PRRSV-ADE infection affects the expression of type II and III IFNs is still unknown. The results of the current study show a suppressive effect of IFN-γ, IFN-λ1, IFN-λ3, and IFN-λ4 by PRRSV-ADE infection in PAMs. By contrast, the production of TGF-β1 in PAMs increased after PRRSV infection *via* the ADE pathway. Moreover, compared with PRRSV+PNI-infected PAMs, a notable transcription decrease in ISG15, ISG56, and OAS2 is seen in PRRSV+PPI-infected PAMs. These results suggest the inhibition of IFN-γ/λs antiviral responses of host cells through the ADE of PRRSV infection. Early studies demonstrated that type I-III IFNs show powerful anti-PRRSV effects (49, 50). Thus, it is not surprising that PRRSV may take advantage of the ADE mechanism to antagonize the activation of these IFNs in target cells for survival. We also observed that PRRSV-ADE infection visibly cut down the transcripts of IRF-3, IRF-7, and NF-κB in PAMs in the early stage of infection (after 12–24 h infection) (Supplementary Figures 1A, B), implying that disruption of these three transcription factors may play a crucial part in PRRSV-ADE infection. In summary, our findings indicate that the ADE pathway of PRRSV infection represses innate antiviral immunity by downregulating the levels of type II and III IFNs, thereby enhancing viral replication. The detailed signaling pathway through which ADE inhibits the production of IFNs remains to be further elucidated in future studies, which will be critical for an in-depth understanding of the molecular mechanisms of ADE-mediated innate antiviral immunosuppression.

## Conclusions

PRRSV alone could induce an antiviral response by upregulating the secretion of type II and III IFNs in PAMs in early infection. Moreover, *via* the ADE pathway, PRRSV suppressed antiviral immunity by downregulating the synthesis of type II and III IFNs in PAMs at any time point postinfection, thereby enhancing viral replication. The ADE mechanism described in this study facilitated our understanding of the pathogenesis of PRRSV-persistent infection.

## Data availability statement

The original contributions presented in the study are included in the article/Supplementary material, further inquiries can be directed to the corresponding authors.

## Author contributions

LZ, XF, and HW are the primary investigators of this study and carried out the data analysis. SH provided suggestions. HF and DL supervised this study. LZ wrote the manuscript. All authors have read and approved the submitted manuscript.
