# Supplementary material for: Antibody-dependent enhancement of porcine reproductive and respiratory syndrome virus infection downregulates the levels of interferon-gamma/lambdas in porcine alveolar macrophages in vitro
Source: Front Vet Sci. 2023 Mar 15;10:1150430. doi: 10.3389/fvets.2023.1150430 (PMC10050554; doi:10.3389/fvets.2023.1150430)
Supplement: Supplementary file 1 [file Data_Sheet_1.DOCX]

Supplementary Material

Antibody-dependent enhancement of porcine reproductive and respiratory syndrome virus infection downregulates the levels of interferon-gamma/lambdas in porcine alveolar macrophages in vitro

Liujun Zhang, Xing Feng, Huandi Wang, Shaojun He, Hongjie Fan*, Deyi Liu*

*** Correspondence:** Hongjie Fan: [[fanhj@ahstu.edu.cn](mailto:fanhj@ahstu.edu.cn);](mailto:email@uni.edu;) Deyi Liu: [liudy@ahstu.edu.cn](mailto:liudy@ahstu.edu.cn)

# Supplementary Figures


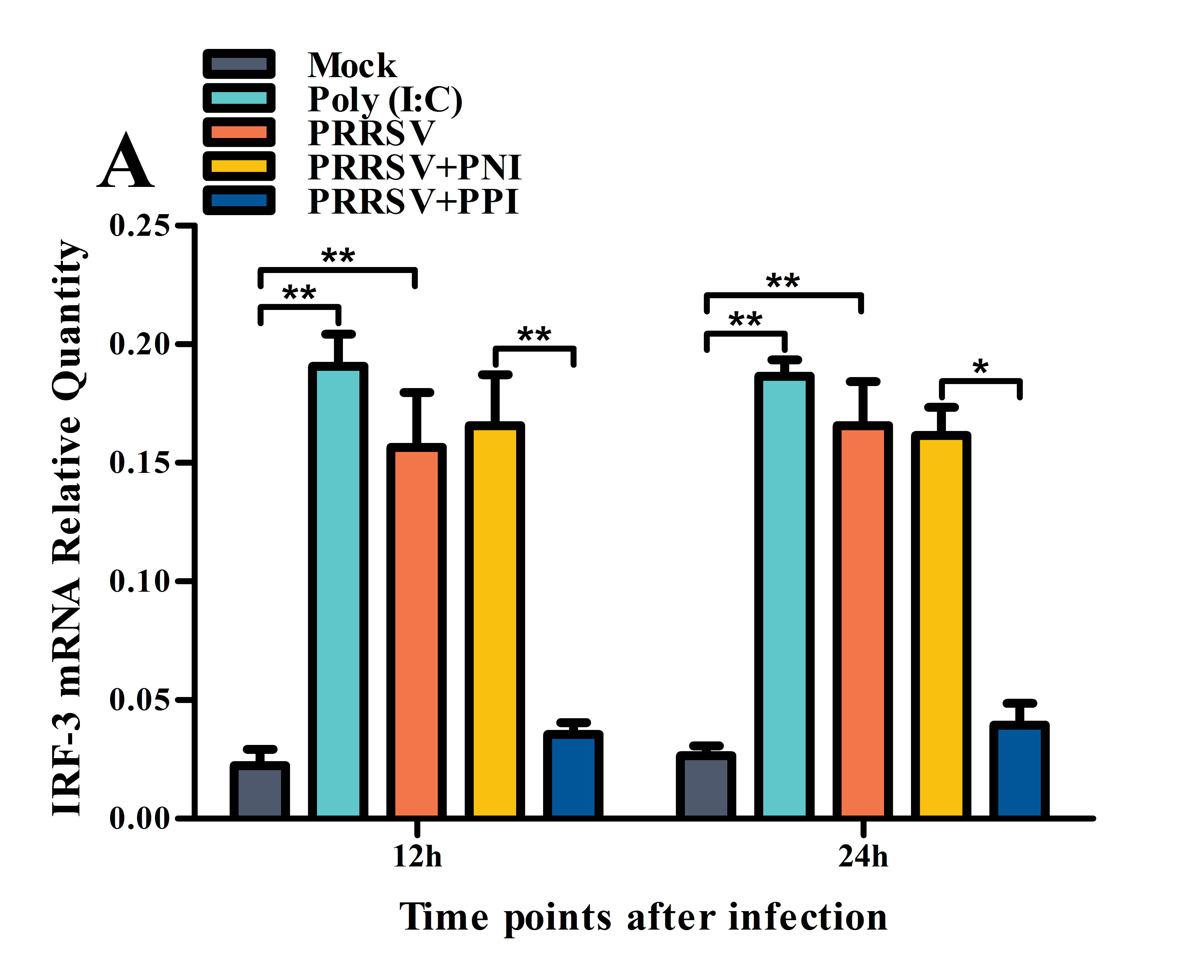

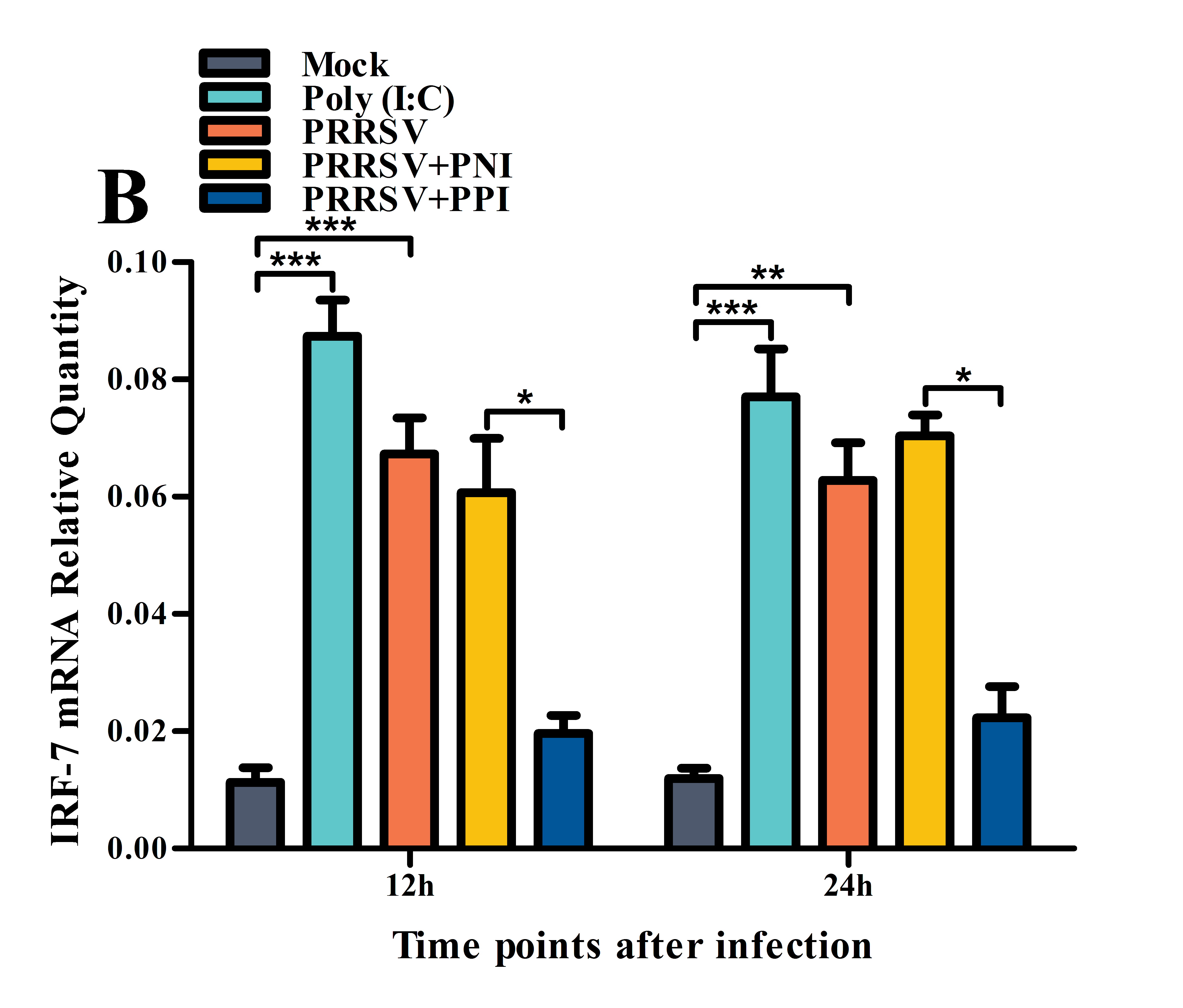

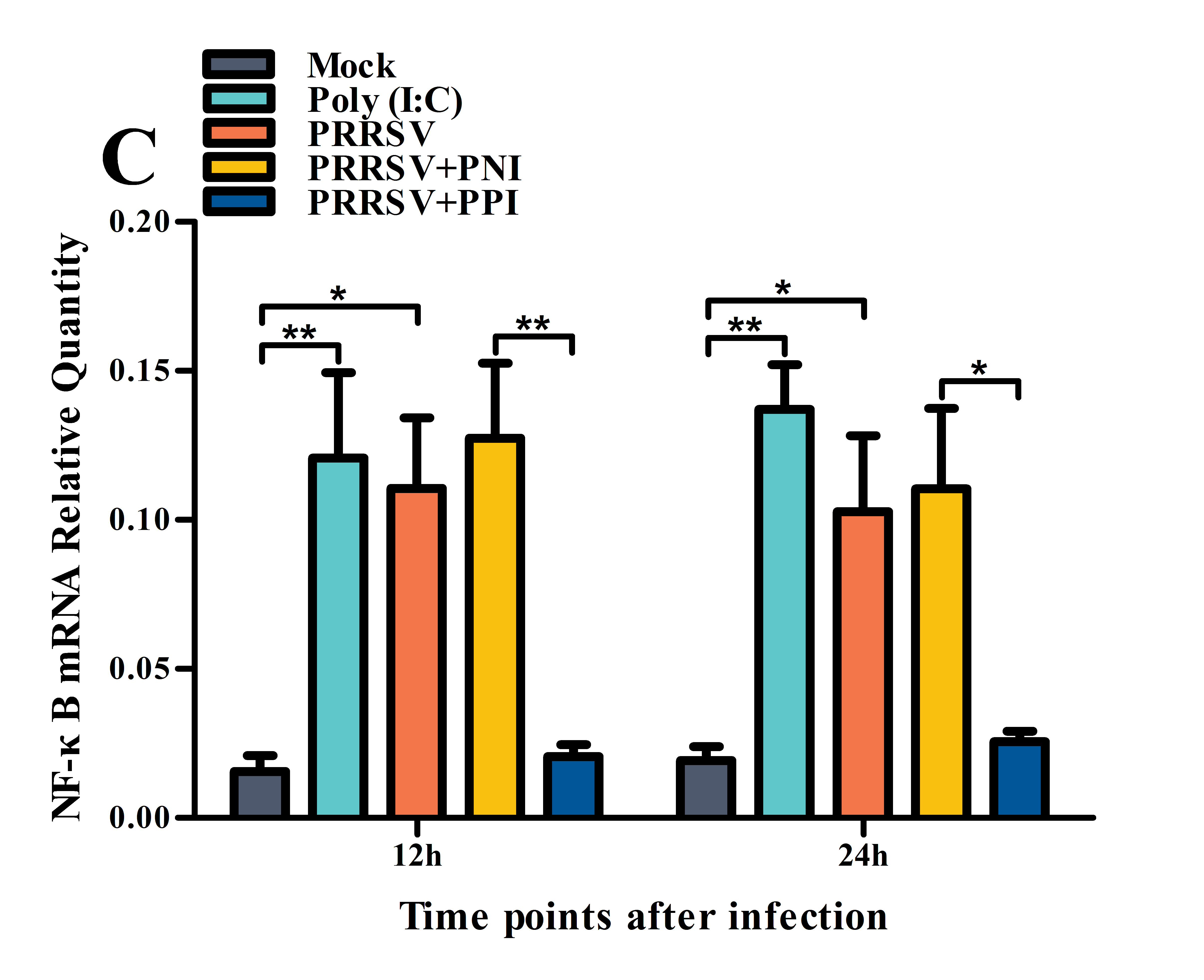


**Supplementary FIGURE S.** The effect of PRRSV or PRRSV-ADE on mRNAs of interferon regulatory factors (IRFs) and nuclear factor kappa-B (NF-κB) in PAMs. The mRNAs of IRFs and NF-κB in treated PAM cells were detected using relative quantitative RT-PCR. **(A)** IRF-3 mRNA; **(B)** IRF-7 mRNA; and **(C)** NF-κB mRNA. The bars indicate the relative expression levels of mRNAs of IRFs or NF-κB. The error bars indicate the SEM from three independent experiments. *** *p* < 0.001, ** *p* < 0.01, and * *p* < 0.05.
